# How much do adverse childhood experiences contribute to adolescent anxiety and depression symptoms? Evidence from the longitudinal study of Australian children

**DOI:** 10.1186/s12888-024-05752-w

**Published:** 2024-04-17

**Authors:** Berhe W. Sahle, Nicola J. Reavley, Amy J. Morgan, Marie Bee Hui Yap, Andrea Reupert, Anthony F. Jorm

**Affiliations:** 1 Centre for Mental Health, Melbourne School of Population and Global Health, University of Melbourne, 207 Bouverie Street, Carlton, Melbourne, VIC 3010 Australia; 2 School of Psychological Sciences and Turner Institute for Brain and Mental Health, Monash University, Melbourne, VIC Australia; 3 Faculty of Education, Monash University, Melbourne, VIC Australia

**Keywords:** Childhood adversity, Adolescent, Anxiety disorder, Depression disorder

## Abstract

This study aims to: (i) examine the association between adverse childhood experiences (ACEs) and elevated anxiety and depressive symptoms in adolescents; and (ii) estimate the burden of anxiety and depressive symptoms attributable to ACEs.

Data were analyzed from 3089 children followed between Waves 1 (age 4–5 years) and 7 (16–17 years) of the Longitudinal Study of Australian Children. Logistic regression was used to estimate the associations between ACEs and child-reported elevated anxiety and depressive symptoms at age 16–17. Anxiety and depressive symptoms were measured using the Children’s Anxiety Scale and Short Mood and Feelings Questionnaire, respectively. The punaf command available in STATA 14 was used to calculate the population attributable fraction (PAF).

Before the age of 18 years, 68.8% of the children had experienced two or more ACEs. In the analysis adjusted for confounding factors, including co-occurring ACEs, both history and current exposure to bullying victimisation and parental psychological distress were associated with a statistically significant increased likelihood of elevated anxiety and depressive symptoms at age 16–17. Overall, 47% of anxiety symptoms (95% CI for PAF: 35–56) and 21% of depressive symptoms (95% CI: 12–29) were attributable to a history of bullying victimisation. Similarly, 17% (95% CI: 11–25%) of anxiety and 15% (95% CI: 4–25%) of depressive symptoms at age 16–17 years were attributable to parental psychological distress experienced between the ages of 4–15 years.

The findings demonstrate that intervention to reduce ACEs, especially parental psychological distress and bullying victimisation, may reduce the substantial burden of mental disorders in the population.

**Supplementary Information:**

The online version contains supplementary material available at 10.1186/s12888-024-05752-w.

## Introduction

Mental disorders remain a major cause of morbidity, mortality, and economic burden worldwide [1, 2]. The lifetime prevalence of having one or more mental disorders by the age of 75 years is estimated to be up to 47% [3]. Despite an increase in the availability of treatment in many countries, there is little evidence that the burden of mental illness is decreasing [4–6]. Moreover, the global economic burden of mental disorders is predicted to rise to $16 trillion by 2030, primarily due to early onset of mental illness and lost productivity across the life course [4].

There is growing evidence that the lack of emphasis on prevention and early intervention underlies the lack of improvements in the population burden of mental disorders [5, 6]. While increasing access to mental health services is central to improving population mental health, even if all those requiring treatment obtained it, approximately 60% of the burden of mental disorders would not be averted [7]. This underscores the importance of prevention of mental disorders with accumulating evidence pointing to the benefits of preventive interventions that aim to reduce risk factors and enhance protective factors [1, 5]. Making progress in this area requires us to target the biggest contributors to mental disorders in order to have a major impact on the population prevalence and burden of disease [8]. These include adverse childhood experiences (ACEs) [8].

ACEs are defined as exposures to traumatic experiences during childhood (0–17 years). They include childhood maltreatment, maladaptive parenting practices (e.g., harsh discipline, aversiveness, over-involvement or parent-child conflict), household dysfunction (e.g., substance or alcohol misuse, family violence, and parental separation/divorce), violence and socio-economic adversity [9, 10]. Globally, ACEs are prevalent, with three in five adults having experienced at least one ACE and a quarter of adults having experienced at least three [11–13]. ACEs are associated with an increased prevalence of physical and mental health problems across the life course, including mental health disorders, suicidal behaviours, unhealthy lifestyle behaviours and chronic non-communicable diseases [14, 15]. For example, children exposed to four or more ACEs have four times higher odds of having anxiety or depressive disorders, compared with children who were not exposed to any ACEs [14]. Furthermore, there is evidence that ACEs and their negative effects can be transmitted from one generation to the next, leading to their intergenerational transmission [16]. In this study we defined ACEs as stressful and potentially traumatic events occurring in childhood or adolescence that can negatively impact health and well-being. ACEs include financial hardship, family drug or alcohol abuse, marital separation, verbal or physical interpersonal conflict, unsafe neighbourhood, parental psychological distress, death of family member, and bullying victimisation [14, 17].

ACEs are common globally, however, the prevalence of specific types of ACEs and their contribution to the risk of mental disorders varies across and within populations [15, 18]. Therefore, intervention efforts need to prioritise those ACEs with the largest potential population benefits in terms of preventing poor mental health outcomes. Calculating the population attributable fraction (PAF) of each ACE can inform action in this area. The PAF combines the prevalence of a risk factor and the strength of association with an outcome, allowing us to measure the proportion of an outcome that would have been prevented in a population over a given period of time by reducing the population’s exposure to a risk factor to a theoretically minimal risk.

Previous studies on the association between ACEs and common mental disorders have a number of limitations, as they are largely focused on individual types of ACEs, such as child maltreatment or bullying [18–21] This approach has been employed despite the frequent co-occurrence of multiple forms of ACEs in certain families, which can have an additive or multiplicative impact on a range of health outcomes. Second, most of the studies examining the links between ACEs and mental disorders involve adults and are based on cross-sectional designs or retrospective assessment of exposure to ACEs in childhood [14, 15]. In addition to the inherent limitations of retrospective assessment, onset of mental disorders in adulthood is likely to be confounded by exposure to a wide range of life events after childhood. Prospective longitudinal studies that include exposure to multiple ACEs across childhood are required to better understand the PAF of ACEs on mental disorders.

Using data from the Longitudinal Study of Australian Children (LSAC), this study examines the association between a range of ACEs and elevated depression and anxiety symptoms, in order to estimate the PAF of anxiety and depressive symptoms associated with ACEs. The LSAC is a large, community-based cohort of Australian children that investigates the effect of children’s social, economic and cultural environments on their wellbeing over the life course [22]. The LSAC provides an excellent opportunity to identify which ACEs are associated with the largest burden from mental disorders in the Australian population.

## Methods

### Data source

This study analysed seven waves of data from the LSAC. Details of the study design, sampling, recruitment, and data collection for LSAC have been described previously [22]. LSAC commenced in 2004 (Wave 1) with a nationally representative sample of 10,090 children drawn from two cohorts of Australian children. Cohort B (“Birth”) includes 5107 children aged 3–19 months and Cohort K (“Kindergarten”) includes 4983 children aged 4 to 7 years at Wave 1. Data have been collected biennially. Overall, LSAC collects data from multiple informants, including children, parents, teachers and childcare workers. Variables collected include family demographics, finances, health status, health behaviour and risk factors, relationships, parenting, long-term chronic conditions, and children’s social and emotional outcomes, via face-to-face interview, self-administered questionnaire, child self-report interview, computer-assisted telephone interview, and observations made by interviewers.

### Measures

We analysed ACEs reported between Waves 1 and 7 of the K-cohort of the LSAC survey, and anxiety and depressive symptoms reported by the child at Wave 7. Our analyses focus on children’s self-reported anxiety and depressive symptoms in the most recent wave because children are deemed to become more reliable reporters of their own mental health as they get older [23].

Anxiety symptoms were assessed based on the 8-item Children’s Anxiety Scale (CAS-8) derived from the Spence Children’s Anxiety Scale short form. Children are asked to rate on a 4-point scale (1 = Never, 4 = Always), the frequency with which they experience symptoms of anxiety such as: ‘I worry about things’; ‘I feel afraid’; and ‘I feel nervous’. The CAS-8 has demonstrated good reliability as an indicator of anxiety symptoms. Total scores of ≥ 13 for males and ≥ 16 for females are considered indicative of elevated or clinical levels of anxiety [24].

Depressive symptoms were assessed using the Short Mood and Feelings Questionnaire (SMFQ). The SMFQ is a 13-item self-report measure of depressive symptoms for children aged 8–16 years. Assessed over the last two weeks, items include, ‘I felt miserable or unhappy’, ‘I didn’t enjoy anything at all’, and ‘I felt I was no good at all’. Response options are true (= 2), sometimes true (= 1), and not true (= 0). Total SMFQ scores range from 0 to 26, and a score of 11 or higher has been shown to have a high sensitivity and specificity in identifying those who meet criteria for a diagnosis of major depressive disorder [25]. In this study, scores of 11 or higher were considered indicative of elevated depressive symptoms.

In each Wave of the LSAC, parents were asked six questions relating to their experience of stressful financial events that occurred in the year preceding the survey. A count of the number of stressful financial events (0–6) was used to indicate the extent of financial hardship, with higher values indicating higher levels of financial stress. Financial stress was dichotomized as having parent 1 and/or parent 2 have at least 1 financial stress vs. neither parent has financial stress.

Parental psychological distress was assessed for each parent at all Waves using the 6-item Kessler Psychological Distress Scale (K-6). Parents reported on a five-point rating scale the extent to which they experience symptoms of psychological distress, such as feeling nervous, hopeless, restless, extremely sad, and worthless over the previous four weeks. Responses were summed and a cut-off point of 13 and above was used for the assessment of probable clinical-level psychosocial distress [26]. Parental psychological distress was dichotomized as: parent 1 and/or parent 2 have psychological distress, or neither parent has psychological distress.

Hostile parenting, which refers to parenting behaviour that expresses hostility, aggression, irritability, and anger towards a child, was assessed through reports by both parents in Waves 3 and 4 [27]. Hostile parenting behaviours were reported on a frequency rating scale (never/almost never; rarely; sometimes; often; always/almost always) to a battery of 4-questions relating to how parents had been feeling or behaving with the child during the preceding four weeks. Item scores were averaged to give overall scores for hostile parenting with higher values indicating higher levels of hostile parenting. Hostile parenting was dichotomized as: parent 1 and/or parent 2 have hostile parenting score in the top 10% vs. neither parent has hostile parenting score in the top 10% [28].

Bullying victimisation between Waves 1 and 4 was reported by the child’s mother and is based on a single question asking whether the child has been picked on or bullied by other children. At Waves 5, 6, and 7, children were asked (on a 4-point rating scale) the following question to assess whether they have experienced bullying: Please indicate if any of the following statements happened during the past 30 days at school: (i) Kids hit or kicked me on purpose; (ii) Kids grabbed or shoved me on purpose; (iii) Kids threatened to hurt me or take my things; (iv) Kids said mean things to me or called me names; (v) Kids tried to keep others from being my friend; (vi) Kids did not let me join in what they were doing; (vii) Kids sent me a mean text message/email; or posted mean things about me on the Internet. Those children who responded ‘never’ to all seven questions were categorised as “not victims of bullying” or otherwise as “bullying victims”.

Verbal inter-parental conflict was assessed in all Waves by asking mothers to rate on a 5-point scale about how often they and their partners engage in disagreements (e.g. ‘‘How often is the conversation awkward or stressful?”). Verbal inter-personal conflict was present if mothers responded “often” or “always” to at least one of the four items [29].

Physical inter-parental conflict was measured at all Waves, by asking mothers to rate on a 5-point scale “How often do you have arguments with your partner that end up with people pushing, hitting, kicking or shoving?’’. A response of “sometimes’’, ‘‘often’’ or ‘‘always’’ represented presence of physical inter-parental conflict [29].

Parent alcohol or substance use problem was assessed by asking the mother if either of the parents had an alcohol or drug problem (Yes/No) in the last year.

Unsafe neighbourhood was defined as “disagreement” or “strong disagreement” with the statement “This is a safe neighbourhood”.

### Data analysis

We estimated the prevalence of the ACEs across the seven Waves, and the prevalence of elevated anxiety and depressive symptoms at Wave 7. We then used logistic regression models to estimate the Odds Ratios (ORs) ± 95% Confidence Interval (CI) of having elevated anxiety or depressive symptoms among those who experienced ACEs compared to those who did not. We ran two separate regression models to compare the: (i) cross-sectional associations between ACEs and elevated anxiety and depressive symptoms at age 16–17 (Wave 7), and (ii) history of exposure to ACEs between ages 4–15 (Waves 1–6) and elevated anxiety and depressive symptoms at age 16–17. In our analyses, where we explored the associations between the prior history of ACEs and depressive and anxiety symptoms at Wave 7, we excluded individuals with elevated anxiety and depressive symptoms in the preceding wave (Wave 6). A cumulative ACE score was calculated based on report of the first exposure to individual ACEs across any of the six follow up waves (1 = yes, 0 = no), and then grouped into categories: 0, 1, 2, and 3 or more. PAFs for anxiety and depressive symptoms due to ACEs significantly associated (*P* < 0.05) with elevated anxiety and depressive symptoms were estimated based on the respective prevalence rates of ACEs and the ORs. The punaf command available in STATA 14 was used to calculate the population attributable fraction (PAF) from the final multivariable logistic regression model. Ethics approval was not required for this because it uses de-identified publicly available data from the LSAC survey.

## Results

### Characteristics of study participants

A total of 3089 children (51% males) responded to Wave 7 of the LSAC survey and were included in this study. Aged 16–17 years, most children (95.9%) were born in Australia and only 9.8% spoke a language other than English at home. Table 1 presents a summary of sociodemographic and other background characteristics of the study population.

**Table 1:** Demographic characteristics of Wave 7 of the K-cohort of the Longitudinal Study of Australian Children

Variable	Categories	N (%) or M ± SD
Sex, males		1576 (51.0)
Socio-Economic Indexes for Areas, bottom 10%		452 (14.6)
Housing	Being paid off by parents	1781 (58.9)
	Owned outright	640 (21.2)
	Rented	536 (17.7)
	Other	65 (2.2)
Mother’s education	Bachelor/postgraduate	814 (41.1)
	Diploma/certificate	1088 (54.9)
	Other	80 (4.0)
Father’s education	Bachelor/postgraduate	1140 (46.0)
	Diploma/certificate	1269 (51.3)
	Other	66 (2.7)
Mother’s employment status	Employed	2526 (84.6)
	Unemployed	63 (2.1)
	Not in labor force	402 (13.3)
Father’s employment status	Employed	2267 (92.6)
	Unemployed	48 (2.0)
	Not in labor force	132 (5.4)
Household income, weekly		$2870 ± 1939
Child’s country of birth	Australia	2962 (95.9)
	Other	127 (4.1)
Mother’s country of birth	Australia	3745 (77.2)
	New Zealand	129 (2.7)
	United Kingdom	271 (5.6)
	Other	703 (14.5)
Father’s country of birth	Australia	3129 (74.9)
	New Zealand	122 (2.9)
	United Kingdom	284 (6.8)
	Other	645 (15.4)
Language spoken at home	English	2762 (90.2)
	Other	300 (9.8)

### Prevalence of ACEs and elevated anxiety and depressive symptoms

Before the age of 18 years, 68.8% of the children had experienced two or more ACEs. Bullying victimisation (54.1%) and exposure to verbal or physical interparental conflict (23.4%) were the most commonly reported ACEs. About a quarter of the parents (23.4%) had experienced two or more (2.1%) financial stresses (e.g., could not pay mortgage or rent on time) and 13.8% had psychological distress. Children had a mean of 2.4 ACEs (SD = 1.3) across the seven Waves. The number of ACEs was comparable in both males and females, but was higher in children of parents who were unemployed or who lived in disadvantaged areas. Table 2 and Supplementary File Table S1) show the prevalence of ACEs between Waves 1 and 7 of the LSAC.

**Table 2:** Prevalence of adverse childhood experiences in the K-cohort of the Longitudinal Study of Australian Children

	Wave 1	Wave 2	Wave 3	Wave 4	Wave 5	Wave 6	Wave 7	Waves 1–7^#^
N	4,983	4,464	4,331	4,169	3,956	3,537	3,089	
Adversity Age	4–5 yrs	6–7 yrs	8–9 yrs	10–11 yrs	12–13 yrs	14–15 yrs	16–17 yrs	
Financial hardships (%)								
0	69.4	81.6	82.7	82.3	82.4	83.5	92.0	65.4
1	16.6	11.3	10.1	10.5	10.9	10.1	5.9	11.5
≥ 2	14.0	7.1	7.2	7.2.	6.7	6.4	2.1	23.1
Parental psychological distress	6.5	5.0	5.6	6.1	6.5	6.9	8.2	13.8
Parent alcohol/drug abuse	4.4	2.0	2.3	3.1	3.5	3.4	5.3	12.0
Verbal interparental conflict (IPC)	6.6	5.8	6.1	5.6	6.3	6.0	6.6	22.6
Physical IPC	0.8	0.8	0.9	0.6	0.9	1.0	1.0	3.0
Verbal or physical IPC	6.9	6.1	6.3	5.7	6.4	6.3	6.8	23.4
Marital separation	2.8	1.8	1.9	4.4	4.4	3.5	4.0	15.3
Unsafe neighbourhood	8.4	4.1	5.6	4.6	2.7	3.6	3.0	18.0
Death of family member	2.7	3.2	3.5	5.7	6.9	6.6	7.7	21.6
Bullying victimization	20.2	31.1	33.0	29.4	41.7	41.0	36.1	54.1
Hostile parenting		10.7	10.4					17.0
Number of ACEs								
0								5.7
1								25.6
2								28.1
3								19.8
≥ 4								20.8

#: cumulative prevalence across the 7 waves

30% of children reported elevated depressive symptoms and 16.1% reported elevated anxiety symptoms at age 16–17 years. The prevalence of both elevated depressive symptoms (36.4% vs. 26.6%) and elevated anxiety (17.6% vs. 14.5%) symptoms was higher in females than males.

### Cross-sectional association between ACEs and anxiety and depressive symptoms

Table 3 shows the cross-sectional association between ACEs and elevated anxiety and depressive symptoms at Wave 7 and the corresponding PAF. After adjusting for potential confounding factors, elevated anxiety and depressive symptoms were significantly higher in children who reported being bullied by other children, and children whose parents experienced psychological distress. Children who reported being bullied by other children (OR = 2.91, 95% CI: 2.23–3.80) and whose parents had psychological distress (OR = 1.90, 95% CI: 1.20–2.99) had greater odds of having elevated anxiety symptoms. Similarly, bullying victimisation (OR = 1.76, 95% CI: 1.28–2.41) and parental psychological distress (OR = 1.86, 95% CI: 1.23–2.79) were independently associated with increased odds of elevated depressive symptoms. Furthermore, a larger total number of ACEs experienced by children was associated with greater odds of elevated depressive or anxiety symptoms. The odds of elevated anxiety symptoms were 2.27, 3.69 and 4.88 times higher in children who reported one, two and three or more ACEs, respectively, compared to those who reported no ACEs. Similarly, children who reported one, two, and three or more ACEs had 1.52, 2.02 and 2.76 times greater odds of elevated depressive symptoms. The association between several other ACEs, including household alcohol or drug abuse, unsafe neighbourhood, and household financial stress, and elevated anxiety and depressive symptoms did not persist after adjusting for potential confounding factors.

**Table 3:** Cross-sectional association between adverse childhood experiences and anxiety and depressive symptoms reported in Wave 7 in the K-cohort

Adversity	Anxiety symptoms					Depression symptoms				
	Crude OR (95% CI)	*p*	Adjusted OR (95% CI)	*p*	PAF	Crude OR (95% CI)	*p*	Adjusted OR (95% CI)	*p*	PAF
Household financial hardship	1.10 (0.88–1.37)	0.390	1.07 (0.85–1.35)	0.545		1.33 (1.11–1.58)	0.002	1.32 (0.96–1.83)	0.085	
Household drug or alcohol abuse	1.14 (0.75–1.75)	0.52	0.98 (0.67–1.44)	0.926		1.53 (1.10–2.15)	0.011	0.94 (0.52–1.69)	0.837	
Marital separation	1.03 (0.62–1.70)	0.91	0.89 (0.58–1.35)	0.596		1.44 (0.97–2.13)	0.064	1.28 (0.60–2.77)	0.520	
Verbal or physical IPC	0.90 (0.58–1.37)	0.613	1.17 (0.90–1.55)	0.242		1.41 (1.03–1.94)	0.032	1.04 (0.62–1.76)	0.876	
Unsafe neighborhood	2.91 (1.86–4.56)	< 0.001	1.18 (0.99–1.41)	0.60		2.74 (1.78–4.22)	< 0.001	1.02 (0.871.20)	0.794	
Parental psychological distress	1.85 (1.22–2.80)	0.004	1.90 (1.20–2.99)	0.006	6 (3-9)	2.06 (1.44–2.93)	< 0.001	1.86 (1.23–2.79)	0.003	5 (2-8)
Death of family member	1.12 (0.80–1.61)	0.505	1.15 (0.83–1.59)	0.388		1.16 (0.87–1.56)	0.299	1.08 (0.78–1.49)	0.627	
Bullying victimization	3.16 (2.48–4.04)	< 0.001	2.91 (2.23–3.80)	< 0.001	47 (36–56)	2.26 (1.90–2.69)	< 0.001	1.76 (1.28–2.41)	< 0.001	21 (12-28)
Any ACEs										
0	Ref		Ref*			Ref		Ref*		
1	2.27 (1.71–3.02)	< 0.001	2.28 (1.69–3.09)	< 0.001		2.22 (1.80–2.74)	< 0.001	1.52 (1.09–2.13)	< 0.013	
2	3.69 (2.64–5.15)	< 0.001	3.50 (2.45–5.03)	< 0.001		3.04 (2.33–3.97)	< 0.001	2.02 (1.38–2.94)	< 0.001	
≥ 3	4.88 (3.02–7.88)	< 0.001	4.10 (2.41–6.99)	< 0.001	47 (34–57)	4.73 (3.10–7.21)	< 0.001	2.76 (1.66–4.60)	< 0.001	33 (15–47)

OR: odds Ratio. PAF: Population attributable fraction; IPC: interparental conflict

Crude OR: adjusted for child’s sex

Adjusted OR: adjusted for child’s sex, other ACEs, employment (parent 1 and 2), highest qualification (parent 1 and 2), house ownership, language spoken at home

*Adjusted for child’s sex, employment (parent 1 and 2), highest qualification (parent 1 and 2), house ownership, language spoken at home

Overall, 47% of anxiety symptoms (95% CI for PAF: 40–57) and 21% (95% CI for PAF: 33–45) of depressive symptoms were attributable to bullying-victimisation. A small but significant proportion of anxiety (PAF: 6%, 95% CI: 3–9) and depressive (PAF: 5%, 95% CI: 2–8) symptoms were attributable to parental psychological distress.

### Association between history of ACEs and elevated anxiety and depressive symptoms

We analysed the association between exposure to ACEs between Wave 1 (4–5 years) and Wave 6 (14–15 years) and elevated anxiety and depressive symptoms at Wave 7 (16–17 years) (Table 4). Bullying victimisation (OR = 1.49, 95% CI: 1.06–2.09) and parental psychological distress (OR = 1.84, 95% CI: 1.24–2.75) were associated with a statistically significant increased odds of elevated anxiety symptoms. Similarly, the odds of elevated depressive symptoms were significantly higher for bullying victimisation (OR = 1.78, 95% CI: 1.24–2.56) and parental psychological distress (OR = 1.41, 95% CI: 1.05–1.91). Children who reported two, three and four or more ACEs had 1.34, 2.75 and 2.53 times greater odds of elevated anxiety, compared to those who reported no ACEs. Similarly, children who reported two, three, and four or more ACEs had 1.43, 1.81 and 1.84 times greater odds of elevated depressive symptoms compared to those who reported no ACEs. However, the increased odds of elevated anxiety and depressive symptoms in children exposed to only one ACE did not reach statistical significance. There was no significant interaction between sex of child and ACEs for elevated depressive or anxiety symptoms. The PAFs of anxiety symptoms associated with ACEs ranged from 6% for financial stress to 15% for parental psychological distress. The PAFs of depressive symptoms associated with bullying victimisation and parental psychological distress were 17% and 15% respectively (Table 4).

**Table 4:** Association between adverse childhood experiences prior to Wave 7 and anxiety and depressive symptoms at Wave 7 in the K-cohort

	Anxiety symptoms					Depression symptoms				
Adversity	Crude OR (95% CI)	P	Adjusted OR (95% CI)	P	PAF (95% CI)	Crude OR (95% CI)	P	Adjusted OR (95% CI)	P	PAF (95% CI)
Household financial hardship	1.44 (1.17–1.78)	0.001	1.21(0.93–1.57)	0.160	6 (1-13)	1.49 (1.26–1.77)	< 0.001	1.12 (0.93–1.34)	0.211	
Household drug or alcohol abuse	1.85 (1.41–2.44)	< 0.001	1.03 (0.68–1.54)	0.186		1.63 (1.29–2.06)	< 0.001	1.17 (0.68–1.99)	0.567	
Marital separation	1.53 (1.18–1.97)	0.001	1.04 (0.58–1.84)	0.902		1.48 (1.19–1.83)	< 0.001	1.60 (0.84–3.03)	0.149	
Verbal or physical IPC	1.33 (0.99–1.79)	0.060	1.15 (0.91–1.62)	0.267		1.64 (1.30–2.08)	< 0.001	1.19 (0.80–1.75)	0.383	
Unsafe neighborhood	1.57 (1.22–2.01)	< 0.001	1.44 (0.90–2.30)	0.125		1.57 (1.27–1.92)	< 0.001	1.33 (0.83–2.15)	0.240	
Parental psychological distress	2.26 (1.65–3.09)	< 0.001	1.84 (1.24–2.75)	0.003	15 (4-24)	2.44 (1.88–3.18)	< 0.001	1.78 (1.24–2.56)	0.002	15 (7-21)
Death of family member	1.52 (1.18–1.97)	0.001	1.14 (0.67–1.96)	0.625		1.48 (1.19–1.83)	< 0.001	1.17 (0.72–1.90)	0.516	
Bullying victimization	1.44 (1.16–1.78)	0.001	1.49 (1.06–2.09)	0.020	13 (7–29.0)	1.36 (1.15–1.61)	< 0.001	1.41 (1.05–1.91)	0.023	17 (11-24)
Hostile parenting	1.24 (0.97–1.58)	0.086	1.20 (0.73–1.96)	0.462		1.01 (0.82–1.24)	0.095	1.10 (0.78–1.55)	0.571	
Any ACEs										
0	Ref		Ref *			Ref		Ref *		
1	1.24 (0.84–1.82)	0.270	1.24 (0.84–1.82)	0.073		1.25 (0.83–1.51)	0.433	1.09 (0.81–1.47)	0.539	
2	1.35 (0.92–1.99)	0.118	1.34 (0.91–1.97)	0.004		1.51 (1.13–2.03)	0.005	1.43 (1.06–1.92)	0.018	
3	1.79 (1.20–2.67)	0.004	2.75 (1.17–2.62)	< 0.001		2.04 (1.49–2.78)	< 0.001	1.81 (1.31–2.48)	< 0.001	
≥ 4	2.61 (1.74–3.91)	< 0.001	2.53 (1.67–3.82)	< 0.001	32 (8–50)	2.21 (1.59–3.06)	< 0.001	1.84 (1.31–2.59)	< 0.001	28 (8-41)

OR: odds Ratio. PAF: Population attributable fraction. Crude OR: adjusted for child’s sex

Adjusted OR: adjusted for child’s sex, other ACEs, employment (parent 1 and 2), highest qualification (parent 1 and 2), house ownership, language spoken at home

*Adjusted for child’s sex, employment (parent 1 and 2), highest qualification (parent 1 and 2), house ownership, language spoken at home

Our findings of the associations between ACEs and elevated depressive symptoms did not substantially change when the cut-off for elevated depressive symptoms was defined as the top 10% of the SMFQ score, and anxiety symptoms as CAS-8 of ≥ 18 for males and ≥ 21 for females (Supplementary File Table S2).

## Discussion

Using a large population-based cohort of Australian children, this study explored the prevalence of a comprehensive list of ACEs and their contribution to the risk of anxiety and depressive symptoms in the population. While ACEs were highly prevalent across all demographic characteristics, bullying victimisation and parental psychological distress were the major contributors to elevated anxiety or depressive symptoms independent of demographic characteristics and coexisting ACEs. The findings strengthen evidence that a substantial burden of anxiety and depressive symptoms in adolescence may be preventable through evidence-based interventions targeting bullying victimisation and parental psychological distress.

A key finding of this study is that even though most of the ACEs were associated with anxiety and depressive symptoms in the individual analyses, after adjusting for potential confounding factors including other ACEs, only bullying victimisation and parental psychological distress remained significant. Previous studies have focused on the association between individual ACEs and mental illness, often without accounting for the effect of co-occurring ACEs, even though most children experience multiple ACEs [15, 30]. In those studies focusing on a single type of ACE, it is not possible to assess whether observed associations represent the downstream effect of other ACEs or are linked to other co-occurring ACEs [31]. Differences in the prevalence of ACEs across populations [32], and variations in access to health and social services that could moderate the impact of ACEs on mental disorders [33], may also partly explain why some ACEs were not significantly associated with anxiety and depressive symptoms in those studies.

It has been consistently reported that ACEs are common across all population groups, although the prevalence rates vary across populations and according to the definition of ACEs [32]. Our findings that two out of three Australian children had experienced two or more ACEs before the age of 18 years is comparable with data from previous studies in other countries [32, 34, 35]. A recent meta-analysis of 96 studies reporting the prevalence of ACEs in school-aged youth (≤ 18 years) found that two thirds of youth experience ACEs no matter where they reside across the world [32]. There was no sex difference in the prevalence of ACEs in this study. Despite the importance of disaggregating the prevalence rates of ACEs by population characteristics, the gender-specific prevalence rate of ACEs is not commonly reported in the literature [32].

Our findings of the extent to which bullying victimisation contributes to elevated anxiety and depressive symptoms are in line with the literature [30]. For example, a birth cohort study from United Kingdom found that 29.2% (95% CI:10.9–43.7) of depression diagnosis at age 18 years was attributable to bullying victimisation at the age of 13 years [30]. Findings from the World Mental Health Surveys showed that parental mental illness was strongly associated with a range of mental health problems in offspring, with a PAF of 13% for anxiety disorders and 10% for mood disorders [36]. Although previous studies have found that both bullying victimisation and parental psychological distress contribute to the risk of anxiety disorders and depression in adolescents, the PAFs may vary across studies mainly due to difference in the prevalence rates of bullying victimisation and parental mental illness. A recent global study of more than 317,000 adolescents (12–17 years) from 83 countries found that the prevalence of bullying victimisation varies across countries, ranging between 8% and 45% [37]. Similarly, the burden of psychological distress varies substantially across population groups [38].

In light of the significant burden of anxiety disorders and depression in adolescence, our findings have important implications for policy and health promotion interventions. Given that bullying victimisation and parental psychological distress are the major contributors to elevated anxiety and depressive symptoms in adolescence, intervention programs that show evidence of reducing rates of these ACEs are likely to have substantial population benefits over time. A meta-analysis of 14 randomized clinical trials of anti-bullying school programs found a significant reduction in bullying and improvement in attitudes against bullying [39]. Another meta-analysis of 53 different anti-bullying programs demonstrated that school-based anti-bullying programs result in a 20% decrease in bullying victimisation [40]. However, it has also been demonstrated that these programs have greater impact in younger children and their effectiveness decreases with age [39, 41]. The substantial burden of elevated anxiety and depressive symptoms attributable to parental psychological distress, and existing evidence of clustering of ACEs in families [13, 42], suggest that children whose parents have elevated psychological distress constitute an essential target group for preventive interventions. There is evidence to show that preventive interventions such as mental health treatment for parents, parenting support and family-focused interventions result in small but significant improvements in child mental health outcomes and a reduction in the risk of intergenerational impacts of parental mental illness [43].

This study has some limitations that should be considered when interpreting the findings. One of the major limitations is that data on child maltreatment, a key ACE that is strongly associated with poor mental health outcomes [15], was not collected in the LSAC. Some of the variables included in the analyses, including depressive symptoms, bullying victimisation and hostile parenting, lack validated measures and thresholds for objectively defining risk, and were therefore defined based on commonly used definitions from previous studies. However, sensitivity analyses conducted with a higher threshold level for indicating elevated depressive symptoms largely support these findings. Given the underrepresentation in the LSAC sample of children from families with a lower socioeconomic status, Aboriginal and Torres Strait Islander families, and children born overseas, the current findings may underestimate the association between ACEs and mental disorders in these subgroups of the Australian population. Although ACEs were collected prospectively in each wave, anxiety and depressive symptoms were not assessed in earlier waves, thereby limiting longitudinal analyses.

## Conclusion

In this large population-based cohort study of Australian adolescents, two-thirds of children were reported as having experienced two or more ACEs before age 18 years. Between 13 and 47% of the burden of depressive or anxiety symptoms at age 16–17 years could be attributed to bullying victimisation, and between 6 and 15% to parental psychological distress. The findings suggest that interventions targeting these ACEs, as the major contributors to elevated anxiety and depressive symptoms in adolescence, may reduce the substantial burden of mental disorders in the population.

### Electronic supplementary material

Below is the link to the electronic supplementary material.

**Supplementary Materials 1: Supplementary file Table S1: **Distribution of adverse childhood experiences in Wave 7 of the K-cohort of Longitudinal Study of Australian Children; **Supplementary file Table S2: **Association between adverse childhood experiences prior to Wave 7 and anxiety and depressive symptoms at Wave 7 in the K-cohort

## Data Availability

The authors do not have permission to share data. LSAC data can be requested through an application to the Australian Data Archive Dataverse at the Australian Government Department of Social Services (https://dataverse.ada.edu.au/).
