# Supplementary material for: How much do adverse childhood experiences contribute to adolescent anxiety and depression symptoms? Evidence from the longitudinal study of Australian children
Source: BMC Psychiatry. 2024 Apr 17;24:289. doi: 10.1186/s12888-024-05752-w (PMC11022337; doi:10.1186/s12888-024-05752-w)
Supplement: Supplementary file 1 — Supplementary Materials 1: Supplementary file Table S1: Distribution of adverse childhood experiences in Wave 7 of the K-cohort of Longitudinal Study of Australian Children; Supplementary file Table S2: Association between adverse childhood experiences prior to Wave 7 and anxiety and depressive symptoms at Wave 7 in the K-cohort [file 12888_2024_5752_MOESM1_ESM.docx]

**Supplementary file Table S1: Distribution of adverse childhood experiences in Wave 7 of the K-cohort of Longitudinal Study of Australian Children**

| Characteristics |  | Number of ACEs | | | | X^2^; P-value |
| --- | --- | --- | --- | --- | --- | --- |
|  |  | 0 | 1 | 2 | ≥3 |  |
| Socio-Economic Indexes for areas |  |  |  |  |  |  |
|  | Top 10 % | 30.2 | 56.0 | 12.0 | 1.8 |  |
|  | Bottom 10 % | 28.7 | 43.1 | 20.7 | 7.5 | 51.4; 0.003 |
| Child’s sex | Male | 30.8 | 51.0 | 14.2 | 3.9 |  |
|  | Female | 28.6 | 51.8 | 15.6 | 3.9 | 2.1; 0.545 |
| Location | Metropolitan | 30.0 | 51.2 | 14.9 | 3.8 |  |
|  | Regional* | 29.3 | 51.7 | 15.0 | 3.9 | 3.8; 0.693 |
| House | Being paid off by parents | 29.6 | 52.8 | 14.5 | 3.0 |  |
|  | Owned outright | 38.0 | 49.8 | 10.6 | 1.5 |  |
|  | Rented | 20.0 | 48.3 | 22.5 | 9.1 | 66.8; <0.001 |
|  | Other | 25.8 | 53.2 | 9.7 | 11.3 |  |
| Language | Non-English | 36.4 | 43.4 | 17.8 | 2.3 |  |
|  | English | 29.1 | 52.3 | 14.6 | 4.0 | 11.2; 0.011 |
| Father | Employed | 31.7 | 52.8 | 13.1 | 2.5 |  |
|  | Unemployed | 23.8 | 45.2 | 23.8 | 7.1 | 89.2; <0.001 |
| Mother | Employed | 30.3 | 52.6 | 14.1 | 2.9 |  |
|  | Unemployed | 12.7 | 43.6 | 17.8 | 7.5 | 70.4; <0.001 |

ACEs: Adverse childhood experiences; *Regional location includes remote (n=26) & very remote areas of Australia (n=11)

**Supplementary file Table S2: Association between adverse childhood experiences prior to Wave 7 and anxiety and depressive symptoms at Wave 7 in the K-cohort**

| Adversity | Anxiety symptoms (SDQ >18 for females and >21 for males) | | | | | Depression symptoms (top 10% of SMFQ) | | | | |
| --- | --- | --- | --- | --- | --- | --- | --- | --- | --- | --- |
|  | Crude OR  (95% CI) | *p* | Adjusted OR  (95% CI) | *p* | PAF | Crude OR  (95% CI) | *p* | Adjusted OR  (95% CI) | *p* | PAF |
| Financial hardship | 1.15 (0.92-1.42) | 0.197 | 1.11 (0.89-2.01) | 0.526 |  | 1.45 (1.12-1.88) | 0.004 | 1.01 (0.57-1.78) | 0.769 |  |
| Drug or alcohol abuse | 1.23 (0.82-1.85) | 0.311 | 1.23 (0.69-2.14) | 0.931 |  | 1.24 (0.86-1.77) | 0.237 | 1.06 (0.45-2.49) | 0.893 |  |
| Marital separation | 1.07 (0.67-1.72) | 0.748 | 1.32 (0.93-1.07) | 0.391 |  | 1.42 (1.04-1.95) | 0.027 | 1.00 (0.91-2.55) | 0.852 |  |
| Verbal or physical IPC | 1.01 (0.78-1.20) | 0.314 | 1.09 (0.75-1.59) | 0.647 |  | 0.96 (0.73-1.26) | 0.794 | 1.19 (0.86-1.64) | 0.271 |  |
| Unsafe neighborhood | 2.71 (1.85-4.64) | 0.002 | 1.00 (0.93-1.37) | 0.867 |  | 1.46 (1.07-1.98) | 0.016 | 1.34 (0.69-2.60) | 0.382 |  |
| Parental psychological distress | 1.77 (1.16-2.70) | 0.007 | 1.82 (1.17-2.84) | 0.008 | 11 (2-19) | 1.59 (1.05-2.42) | 0.028 | 1.39 (1.13-2.25) | 0.034 | 10 (4-16) |
| Death of family member | 1.12 (0.69-1.39) | 0.931 | 1.12 (0.78-1.62) | 0.673 |  | 1.24 (0.93-1.66) | 0.133 | 1.14 (0.90-1.61) | 0.202 |  |
| Bullying  victimization | 2.97 (2.40-3.69) | <0.001 | 2.09 (1.55-4.54) | 0.003 | 15 (7-23) | 1.81 (0.78-1.31) | 0.904 | 1.58 (1.15-3.82) | 0.015 | 38 (21-58) |
| Hostile parenting |  |  |  |  |  | 1.09 (0.73-1.63) | 0.360 | 1.08 (0.63-1.84) | 0.768 |  |
| Any ACEs |  |  |  |  |  |  |  |  |  |  |
| 0 | Ref |  | Ref* |  |  | Ref |  |  |  |  |
| 1 | 2.21 (1.72-2.84) | <0.001 | 2.38 (1.53-3.71) | <0.001 |  | 1.21 (0.88-1.65) | 0.235 | 1.05 (0.56-1.97) | 0.864 |  |
| 2 | 3.18 (2.33-4.34) | <0.001 | 2.38 (1.32-4.32) | <0.001 |  | 1.99 (1.36-2.90) | <0.001 | 2.99 (1.39-6.45) | 0.005 |  |
| ≥3 | 4.87 (3.01-7.87) | <0.001 | 4.54 (1.95-10.55) | <0.001 | 39 (20-53) | 2.09 (1.16-3.78) | 0.014 | 3.83 (1.31-11.18) | 0.014 | 28 (12-50) |

OR: odds Ratio. PAF: Population attributable fraction; IPC: interparental conflict

Crude OR: adjusted for child’s sex.

Adjusted OR: adjusted for child’s sex, other ACEs, employment (parent 1 and 2), highest qualification (parent 1 and 2), house ownership, language spoken at home. *Adjusted for child’s sex, employment (parent 1 and 2), highest qualification (parent 1 and 2), house ownership, language spoken at home.
